# Diagnostic challenges for *Aelurostrongylus abstrusus* infection in cats from endemic areas in Italy

**DOI:** 10.1186/s13071-023-05808-y

**Published:** 2023-06-06

**Authors:** Alice Vismarra, Manuela Schnyder, Christina Strube, Laura Kramer, Liliana Colombo, Marco Genchi

**Affiliations:** 1 Department of Veterinary Medicine Sciences, University of Parma, Strada del Taglio, 10, 43126 Parma, Italy; 2 Institute of Parasitology, Vetsuisse Faculty, University of Zurich, Winterthurerstrasse 266a, 8057 Zurich, Switzerland; 3 Institute for Parasitology, Centre for Infection Medicine, University of Veterinary Medicine Hannover, Buenteweg 17, 30559 Hannover, Germany; 4 MSD Animal Health, Via Fratelli Cervi, 20090 Segrate, MI Italy

**Keywords:** *Aelurostrongylus abstrusus*, Serology, Cats, Risk factors, Italy

## Abstract

**Background:**

The lungworm *Aelurostrongylus abstrusus* infects wild and domestic feline species worldwide and is considered a primary respiratory parasite of cats. Definitive diagnosis is based on the identification of first-stage larvae (L1s) released in faeces approximately 5 to 6 weeks after infection. More recently, serology has been shown to be a diagnostic alternative for *A. abstrusus* infection in cats. The present study aimed at evaluating the diagnostic performance of serological antibody detection compared to faecal examination for *A. abstrusus* infection in a population of cats with known infection status from endemic areas in Italy and to identify factors (larval scores, age, co-infections with other helminths) that may influence test sensitivity and specificity of serology.

**Methods:**

All cats resulting positive using the Baermann technique (*n* = 78) were tested with the *A. abstrusus* ELISA. An additional 90 serum samples from cats living in three geographical areas with infection prevalence > 10%, but that resulted negative on Baermann, were also tested.

**Results:**

Among 78 cats copromicroscopically positive for L1s of *A. abstrusus* (Group 1), 29 (37.2%) were seropositive in ELISA. Of the 90 cats from Group 2 (cats living in three geographical areas in Italy with *A. abstrusus* prevalence > than 10%, but negative on Baermann examination), 11 (12.2%) were positive on ELISA. The overall seroprevalence was 23.8%. There was no statistical difference either between average optical density (OD) values of cats excreting > 100 L1s vs. cats excreting < 100 L1s (0.84 vs. 0.66; *P* value = 0.3247) or comparing the OD values with age of infected cats. Few Baermann-negative cats positive for *Toxocara cati* or hookworms were seropositive, supporting lack of cross-reactivity to these nematodes.

**Conclusions:**

Results from the present study suggest that relying solely on faecal examination may underestimate prevalence of *A. abstrusus* infection in cats and that field surveys based on antibody detection are useful for establishing true prevalence of infected and/or exposed animals.

**Graphical Abstract:**

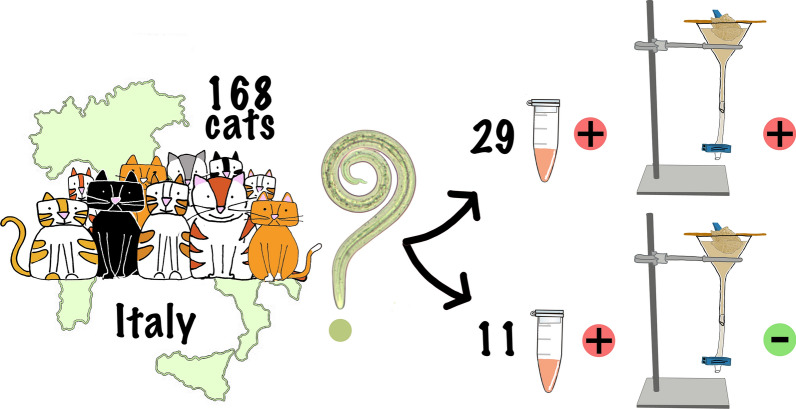

## Background

The lungworm *Aelurostrongylus abstrusus* infects wild and domestic felids worldwide and is considered a primary respiratory parasite of cats [1, 2].

*Aelurostrongylus abstrusus* has an indirect life cycle, with gastropods as intermediate hosts and various vertebrates serving as paratenic hosts (e.g., lizards, frogs, snakes), which are likely the primary source of infection in cats [3]. Outdoor access and hunting behaviour are important risk factors for infection in definitive hosts [4, 5].

Many infected cats are asymptomatic, while others exhibit clinical signs ranging from coughing and anorexia to dyspnoea and tachypnoea, depending on the severity of the infection. In some cases (young cats and cats with high parasite load), infection with *A. abstrusus* can be fatal [6–8].

Definitive diagnosis is based on identification of first-stage larvae (L1s) released in faeces approximately 5 to 6 weeks after infection [9]. The Baermann method, based on the principle of larval migration, is the most frequently used diagnostic technique. Recovered L1s are motile and have a distinct S-shaped kinked tail with a dorsal spine; however, their morphological differentiation from *Troglostrongylus* sp. and *Oslerus rostratus* L1s is challenging [10]. Limits to the sensitivity of this technique include the need for fresh faeces, the possible intermittent or interrupted excretion of larvae, and the impossibility of diagnosing pre-patent infections [9, 11].

More recently, serology has been shown to be a sensitive diagnostic alternative for the detection of *A. abstrusus* infection in cats. Zottler et al. [12] developed an enzyme-linked immunosorbent assay (ELISA) using a recombinant bovine lungworm major sperm protein (MSP) as a detection antigen [13]. The authors reported 100% sensitivity in experimentally infected cats at approximately 70 days post-infection. Sensitivity in naturally infected cats was 88% [12]. Cross-reactions with other helminths were not observed in cats experimentally infected with *Toxocara cati* or *Ancylostoma tubaeforme*, whereas infection of other helminths in naturally infected stray cats resulted in variable rates of apparent cross-reactivity in *A. abstrusus* ELISA: in these cats, coproscopic false-negative *A. abstrusus* infections could not be excluded [12].

The present study aimed at evaluating the diagnostic performance of serology compared to faecal examination for *A. abstrusus* infection in a population of cats with known infection status (based on L1 detection) from endemic areas in Italy and to identify factors (larval scores, co-infections with other helminths) that may influence test sensitivity and specificity.

## Methods

### Animals

Cats from the present study represent a sub-population of cats enrolled in a previous nationwide survey of feline ecto- and endoparasites in Italy [14]. During the previous study, faecal samples and blood were collected from 987 cats from 13 Italian regions. All had outdoor access and had not received any antiparasitic treatment in the 3 months prior to sampling.

Blood samples (2 ml) were stored at − 20 °C. Faecal samples were processed with the Baermann technique (5 g of faeces) and examined for lungworm larvae after 12 h. Larval load was scored as “low” (< 100 L1s per gram of faeces [lpg]) or “high” (> 100 lpg).

Seventy-eight out of 769 cats from 10 regions resulted positive for *A. abstrusus* for an overall prevalence of 10.1%, with values ranging from 1.2 to 22.4%, depending on the geographical area (14).

### Serology

All cats resulting positive in the Baermann technique (*n* = 78) were tested with the *A. abstrusus* ELISA (Group 1). An additional 90 serum samples from cats living in three geographical areas with infection prevalence > 10%, but that resulted negative with Baermann technique, were also tested (Group 2; 30 additional samples from Messina, Naples, and Sassari, respectively). Blood samples were thawed and centrifuged at 1200 × *g* for 10 min to obtain serum.

Serology was carried out according to Gueldner et al. [3] with minor modifications: briefly, Immobilizer Amino Plate (Nunc Roskilde, Denmark) was coated with recombinant MSP diluted in 20 mM phosphate-buffered 150 mM saline (PBS-2, pH 7.4) at a concentration of 0.250 µg MSP/well. After incubation and washing steps, sera diluted 1:200 in PBS-2-Tween (100 µl/well) were added. Following further incubation and washing steps, plates were incubated with an HRP-labeled goat anti-feline IgG (Southern Biotech, Birmingham, AL, USA) at a dilution of 1:9000 in PBS-2-T (100 µl/well). After the final washing steps, the wells were filled with 50 µl/well of σ-phenylene-diamine dihydrochloride (Sigma-Aldrich, St Louis, MO, USA) in 25 mM citrate/50 mM phosphate buffer containing 0.04% hydrogen peroxide and incubated in the dark. The absorbance values were read in a Multiscan RC ELISA reader (Thermo Labsystems, Helsinki, Finland) initially at 450 nm to determine the exact moment for stopping the reaction with sulphuric acid; subsequently, the plate was read at 492 nm. Each plate was run with a substrate control, two positive controls (sera from experimentally infected cats), two negative controls (sera from uninfected laboratory cats) and a conjugate control. A reference serum was added twice on each plate to calculate a correction factor for adjustment between plates [15]. The cut-off value was determined as previously described [16].

### Statistical analyses

The difference between the average optical density (OD) value compared between the groups (< 100 lpg and > 100 lpg) was evaluated with a t-test using the GraphpadPrism 9 software (version 9.4.1 for macOS).

The optical density was analysed using the MIXED procedure (SAS Institute Inc., Cary, NC) according to the following base model:

$${\text{y}}_{{{\text{ij}}}} = \, \mu \, + {\text{ NL}}_{{\text{i}}} + {\text{ e}}_{{{\text{ij}}}} ,$$

where y_ij_ is the observed trait (OD); μ is the overall intercept of the model; NL_i_ is the fixed effect of the number of L1s (i = 2 levels; 1 =  > 100; 2 =  < 100); and e_ij_ is the residual random error term ~ N (0, σ^2^_e_).

A further model (extended model) was used to analyse the effect of the number of lpg 1 s corrected for the age (3 levels; 1 =  ≤ 6 months; 2 = 7–24 months; 3 =  > 24 months) and sex effects (2 levels).

The difference was considered statistically significant when the *P* value was < 0.05 (two-tailed *P* value).

## Results

Tables 1 and 2 summarize the serological results performed on samples from cats positive or negative for *A. abstrusus* infection with the Baermann method.

**Table 1:** Serological results of Group 1: cats previously tested positive for excretion of first-stage larvae of *Aelurostrongylus abstrusus* by the Baermann method and originating from various regions of Italy

Region	BA	BO	CZ	ME	MI	NA	PD	PG	SS	TE	Total
No. cats tested in Baermann	84	14	84	85	84	89	64	89	91	85	769
No. cats Baermann + (%)	3(3.6)	2(14.3)	1 (1.2)	19 (22.4)	4 (4.8)	15 (16.8)	3 (4.7)	5 (5.6)	19 (20.9)	7 (8.2)	78 (10.14)
No. sera ELISA + (%)	0/3 (0)	0/2 (0)	1/1 (100)	7/19 (36.8)	2/4 (50)	2/15 (13.3)	1/3 (33.3)	1/5 (20)	11/19 (57.9)	4/6 (66.6)	29(37.2)

*BA* Bari, *BO* Bologna, *CZ* Catanzaro, *ME* Messina, *MI* Milano, *NA* Napoli, *PD* Padova, *PG* Perugia, *SS* Sassari, *TE* Teramo

**Table 2:** Serological results of Group 2: cats previously tested negative for excretion of first-stage larvae of *Aelurostrongylus abstrusus* by the Baermann method and originating from three selected regions of Italy with prevalence above 10%

Region	ME	SS	NA	Total
N. serum samples tested in ELISA	30	30	30	166
N. sera Baermann −/ELISA + (%)	7/30 (23.3)	3/30 (10)	1/30 (3.3)	11/90 (12.2)

*ME* Messina, *SS* Sassari, *NA* Napoli

Among the 78 cats from Group 1, 29 (37.2% CI 95% = 26.5–48.87) were seropositive for *A. abstrusus* antibodies. Of the 90 cats from group 2, 11 (12.2% CI 95% = 6.26–20.82) were seropositive. The overall seroprevalence was 23.8%.

Table 3 reports the larval scores of Baermann +/ELISA + cats and associated OD values. There was no statistical difference between average OD values of cats excreting > 100 lpg vs. cats excreting < 100 lpg (0.84 vs. 0.66; unpaired t-test *t* = 1.004, *P* value = 0.3247); also comparing the OD values with age and the number of lpg the difference was not significant (ANOVA *F* = 0.78, *P* = 0.386).

**Table 3:** OD values in 29 cats positive for low/high larval scores

	Sample origin	> 100 *A. abstrusus* lpg				Sample origin	< 100 *A. abstrusus* lpg		
		Months	Sex	OD values			Months	Sex	OD values
**1**	CZ015	3	M	0.594	1	ME021	5	M	0.384
**2**	ME005	24	M	0.683	2	ME064	4	M	0.651
**3**	ME036	2	F	0.598	3	MI073	1	M	0.469
**4**	ME050	8	F	1.030	4	MI075	96	F	0.440
**5**	ME055	12	M	0.617	5	NA055	24	M	0.338
**6**	ME074	4	F	0.706	6	NA070	24	F	0.674
**7**	SS026	48	F	0.531	7	PD015	96	M	0.478
**8**	SS032	96	M	2.238	8	PG075	36	M	0.332
**9**	SS043	6	F	0.507	9	SS025	12	M	0.585
**10**	SS044	6	F	2.178	10	SS056	108	F	0.710
**11**	SS058	6	F	0.382	11	SS057	6	F	1.433
**12**	SS065	36	F	0.324	12	SS060	24	F	1.910
**13**	TE004	60	M	0.461	13	SS081	12	F	0.404
	Average value	23.9		0.84	14	TE044	96	M	0.383
					15	TE051	24	M	1.041
					16	TE055	8	M	0.288
						Average value	36		0.66

*CZ* Catanzaro, *ME* Messina, *MI* Milano, *NA* Napoli, *PD* Padova, *PG* Perugia, *SS* Sassari, *TE* Teramo

The non-parametric statistics reported a very low level of correlation (Pearson correlation coefficient calculation) with non-significant *P* values when the age (expressed in months) was compared with OD values recorded by cats in group 1 (> 100 lpg; Pearson correlation, *r* = 0.3497 *P* value: 0.2415) and group 2 (< 100 lpg; Pearson correlation, *r* = − 0.1625 *P* value: 0.5488).

Co-infection with other helminths was common in the study population. Of the 78 cats shedding L1s of *A. abstrusus*, 27 were co-infected with *T. cati* and 14 with hookworms. Twelve of the 27 cats with *T. cati* and 7 of the 14 with hookworms were also seropositive for *A. abstrusus* antibodies. Forty-six of the 90 (51%) Baermann-negative cats tested in ELISA were co-infected with either *T. cati* or hookworms. However, only three of these were seropositive by *A. abstrusus* antibody detection ELISA.

## Discussion

Results of the present study are similar to those from a previous study comparing faecal examination with serology for *A. abstrusus* diagnosis. Morelli et al. [16] evaluated a population of 220 cats from different geographical areas of Greece. Authors reported that 11.8% of cats shedding *A. abstrusus* L1s were also seropositive for *A. abstrusus* antibodies, while 15.5% of cats that were negative on faecal examination were seropositive. Overall seropositivity of the study population was 27.3%. In the present study, the percentage of Baermann-positive/ELISA-positive cats was higher (37.2%), while the percentage of Baermann negative/ELISA positive was similar (12.2%). Overall, seroprevalence of our study population was also in line with the previous study (23.8%). Several other studies evaluating seroprevalence in feline populations with unknown infection status have reported average values ranging from 9 to 22%, depending on geographical regions [3, 17–19]. In contrast, previous studies relying solely on faecal examination for *A. abstrusus* infection [1;4] have reported consistently lower prevalence values, including the study of the larger cat population from which the cats in the present study were chosen [14], which reported 10.1% of infected cats. Clearly, the selection of cats here can be considered as a bias and thus higher prevalence was to be expected.

It has been suggested that faecal examinations underestimate prevalence of *A. abstrusus* in cats and that serology can identify cats that are infected but not shedding L1s. Zottler et al. [12] reported that a portion of experimentally infected cats begin to mount a specific antibody response as early as 20 days post-infection, thus before the onset of patency. Cats may also remain seropositive following resolution of a patent infection, indicating the usefulness of serology for evaluating risk of exposure in each area. However, more importantly, experimental studies also showed that *A. abstrusus*-infected cats may shed L1s intermittently [9] or that non-patent infections also occur in chronically or repeatedly infected cats [11, 20]: in such cases, Baermann technique analyses and other methods relying on the excretion of L1s are insufficient to identify infected cats. Missing cats infected with *A. abstrusus* can be of high relevance in case of anaesthesia, as lung damage may expose them to a higher risk of death during or after anaesthetic procedures [21].

In the present study, a proportion of cats were Baermann positive but negative in serology. This may partially be due to the time post-infection necessary to achieve detectable antibody levels. Zottler et al. [12] reported that 100% sensitivity of the ELISA in experimentally infected cats was observed at approximately 10 weeks post-infection when infections can be already patent for 4 weeks. In an experimental follow-up study single cats became seropositive even 11 and 16 weeks post infection [22]. In addition, for free-roaming cats Morelli et al. [16] suggested that the immune status may also influence ELISA results, for instance when immunosuppressive viruses (FIV, FeLV) could dampen humoral response to *A. abstrusus*.

**Fig. 1:**
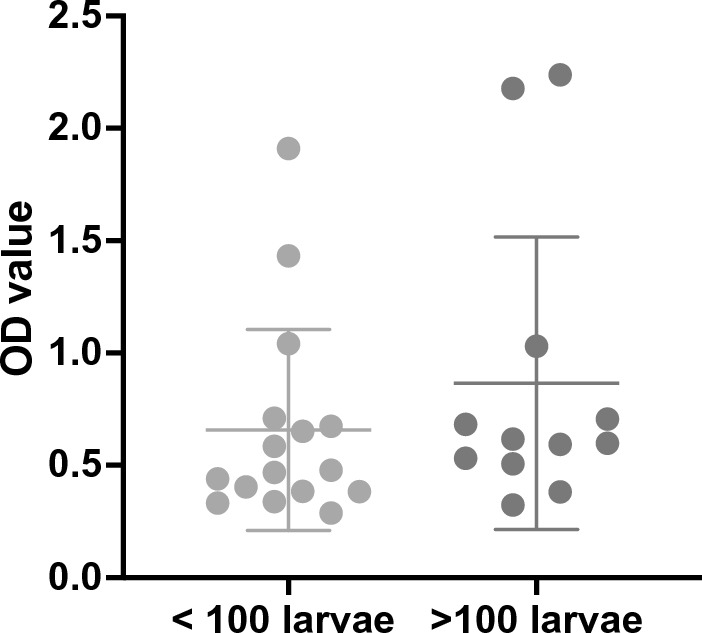
The difference between average OD values compared between the group of cats excreting < 100 *Aelurostrongylus abstrusus* lpg and > 100 lpg was not statistically significant (*P* value: 0.3247)

The development of the antibody detection ELISA used in the present study was based on serum samples from cats infected with 600–800 *A. abstrusus* L3s and with an average adult worm burden of 28; the authors reported that there was no correlation between the number of adult worms detected at necropsy and OD values in ELISA [12]. Strube et al. [23] have also reported similar results in cattle infected with *Dictyocaulus viviparus*, where infection dose was not correlated with serological response to MSP antigen. In the present study, positive OD values ranged from 0.332 to 2.238. Even though there was no statistical association between larval score and average OD values, cats excreting > 100 lpg tended to have higher OD values and approximately 70% of Baermann-positive/ELISA-negative cats had < 100 lpg. It would be interesting to further investigate the correlation between infective dose and antibody response to potentially correlate the sensitivity of serology for *A. abstrusus* with parasite load (Fig. 1).

In an experimental study with six cats infected with 300 L3s, the serological response correlated approximately with the number of L1s excreted in faeces, and it was concluded that L1 production is associated with the presence of many male worms (inducing anti-MSP antibodies) or high MSP release by them [22].

The used recombinant MSP antigen is nematode-specific, preventing cross-reactions with trematodes and cestodes. However, it has been suggested that cross-reactivity with other nematodes in a naturally infected cat population may influence specificity of *A. abstrusus* serology [12], particularly regarding other metastrongyles like *Troglostrongylus* spp. [16, 18]. In the previous study [14], *Troglostrongylus* spp. infections were not identified in faecal samples. In contrast, 65% of Baermann technique-positive/ELISA-positive cats were co-infected with either *T. cati* or hookworms. Cats from Group 2 also had a high co-infection rate with roundworms and/or hookworms (51%), and 6.5% of these resulted positive on ELISA, but very few Baermann-negative cats with *T. cati* or hookworms were positive by ELISA, confirming a lack of cross-reactivity to these nematodes.

## Conclusions

Results from the present study suggest that relying solely on faecal examination may underestimate the prevalence of *A. abstrusus* infection in cats and that field surveys based on antibody detection are useful for establishing the prevalence of infected and/or exposed animals. The marked prevalence of *A. abstrusus* in the present study, and in other geographical areas of Italy, suggest the need for greater attention on the part of practitioners. The use of anthelminthic drugs that are able to prevent the establishment of adult *A. abstrusus* should be considered as part of the overall healthcare protocol of cats living in endemic areas*.*

## Data Availability

All data obtained are shown in the manuscript.

## Abbreviations

*A. abstrusus*: *Aelurostrongylus abstrusus*
L1S: First-stage larvae
OD: Optical density (OD)
ELISA: Enzyme-linked immunosorbent assay
MSP: Major sperm protein of bovine lungworm
LPG: L1s per gram of faeces
FIV: Feline immunodeficiency virus
FeLV: Feline leukemia virus
L3S: Third stage larvae
